# Atrial Fibrillation in a Young Healthy Adult: A Case Report From Anaphylaxis to Arrhythmia

**DOI:** 10.7759/cureus.96295

**Published:** 2025-11-07

**Authors:** Louis A Enchill, Eugene K Yeboah, Sergei Klokov, Ogheneakpobor E Ubogun, Suchith B Suresh

**Affiliations:** 1 Internal Medicine, Montefiore St. Luke's Cornwall Hospital, Newburgh, USA; 2 Internal Medicine, State University of New York Downstate Medical Center, Brooklyn, USA

**Keywords:** anaphylaxis, atrial fibrillation in young adults, atrial fibrillation with rapid ventricular response (rvr), bee sting, drug-induced arrhythmia

## Abstract

Atrial fibrillation (AF) is rare in young adults without underlying cardiac pathology. We present a 33-year-old previously healthy male who developed new-onset AF with rapid ventricular response (RVR) after receiving epinephrine for a bee sting-associated allergic reaction. The arrhythmia occurred within five minutes of intramuscular epinephrine administration and resolved following rate control with intravenous diltiazem. Cardiac workup, including echocardiography and thyroid function tests, was unremarkable. He remained in sinus rhythm during telemetry monitoring and was discharged with outpatient follow-up. This case highlights the potential for transient AF in the setting of allergic reactions and raises diagnostic and therapeutic questions regarding the role of venom-induced autonomic effects versus sympathomimetic drug administration.

## Introduction

Atrial fibrillation (AF) is the most common sustained arrhythmia, with prevalence increasing with age and associated comorbidities such as hypertension, diabetes, coronary artery disease, and valvular heart disease. By contrast, AF is rare in healthy individuals under age 40 without structural heart disease or other risk factors. When it does occur in this population, secondary causes such as drugs, toxins, acute illness, and sympathomimetic agents must be considered [1].

Bee stings, particularly from Hymenoptera, can cause systemic reactions ranging from local inflammation to full-blown anaphylaxis. Bee venom contains biologically active compounds, including melittin, apamin, phospholipases, and histamine-releasing factors that can cause varied physiologic effects [2]. Separately, epinephrine, the cornerstone of anaphylaxis treatment, has well-documented pro-arrhythmic potential, especially via β1-adrenergic stimulation, increasing automaticity and conduction velocity in atrial tissue [3].

This case explores a diagnostic dilemma in a young adult who developed new-onset AF following a bee sting and subsequent administration of epinephrine. What was the likely trigger: the venom or the drug?

## Case presentation

A 33-year-old male with no known medical history presented to the emergency department one hour after sustaining a bee sting to his left lower leg. He reported sudden-onset light-headedness, mild shortness of breath, vertigo, one episode of vomiting, and a localized rash at the sting site. He denied chest pain, palpitations, throat tightness, wheezing, abdominal discomfort, or diarrhea. He reported a previous allergic reaction to a bee sting two years earlier, which was self-limited and did not require hospitalization.

On arrival, his blood pressure was 128/73 mmHg, and his heart rate was 87 beats per minute. His respiratory rate and oxygen saturation were within normal limits. Physical examination revealed localized erythema and rash at the sting site, with no stridor, wheezing, or respiratory distress.

**Figure 1:**
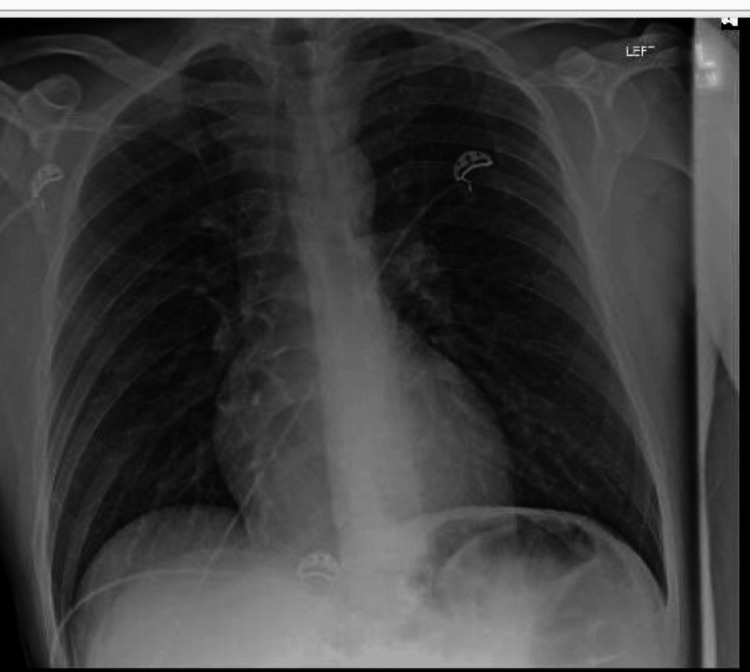
Posteroanterior (PA) view of the patient's chest X-ray

He was treated for presumed anaphylaxis with 0.5 mg of intramuscular epinephrine (one in 1000), 1-liter intravenous normal saline bolus (0.9%) at 2 L/hour, 125 mg intravenous methylprednisolone, 25 mg intravenous diphenhydramine, oxygen therapy via nasal cannula, and nebulized albuterol/ipratropium. Baseline laboratory tests (Table 1) showed neutrophilic leukocytosis, likely due to corticosteroid administration or the anaphylaxis, which resolved upon repeat the next day, dropping from 18.29 k/UL to 7.47 k/UL. Chest X-ray (Figure 1) and basic metabolic panel were unremarkable.

**Table 1:** Results of the laboratory tests carried out on the patient at presentation.

Laboratory tests (blood)	Result	Reference range
Comprehensive metabolic panel		
Sodium	136	(135-145) mmol/l
Potassium	4.2	(3.5-5.1) mmol/l
Chloride	108	(98-107) mmol/l
Creatinine	0.847	(0.7-1.3) mg/dl
Blood urea nitrogen (BUN)	14	(7-18) mg/dl
Calcium	9.7	(8.5-10.1) mg/dl
Magnessium	1.9	(1.8-2.4) mg/dl
Glucose	156	(74-106) mg/dl
Alkaline phosphatase	77	(45-117) U/L
Aspartate aminotransferase	23	(15- 37) U/L
Alanine aminotransferase	36	(16-61) U/L
Albumin	3.6	(3.4-5.0) g/dL
Complete blood count		
White blood cell count	18.3 (89.3% neutrophils)	(4-10) kU/L
Hemoglobin	16.3	(13.7-17.5) g/dL
Mean corpuscular volume (MCV)	85.3	(80-96) fL
Mean corpuscular hemoglobin (MCH)	28.4	(32.2-35.5) pg
Platelet count	187	(150-450) kU/L
Troponin I (HS)	4.2	(3-78.0) ng/L
Thyroid-stimulating hormone	0.82	(0.358- 3.740) uIU/mL

**Figure 2:**
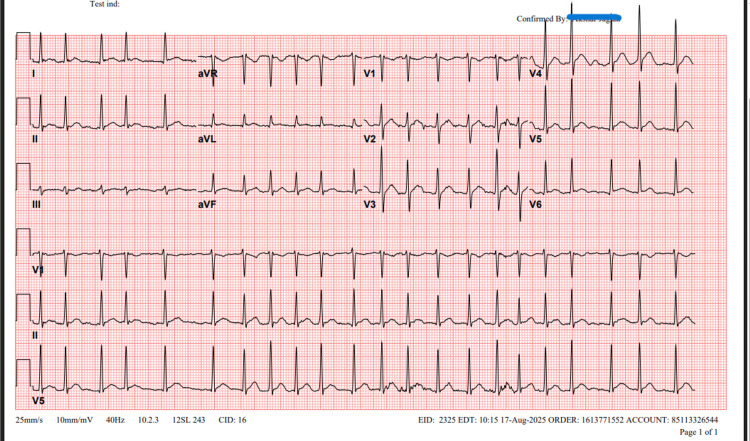
Electrocardiogram (ECG) of the patient showing atrial fibrillation with rapid ventricular response

Approximately five minutes after epinephrine administration, telemetry monitoring revealed new-onset tachycardia. Electrocardiogram confirmed AF with rapid ventricular response at 132 beats per minute (Figure 2). Initial treatment with 20 mg intravenous diltiazem and subsequently 5 mg intravenous metoprolol boluses was ineffective at achieving sustained rate control. A continuous diltiazem infusion (125 mg in 125 ml of normal saline) at 10 mg/ hour was initiated, resulting in successful rate control and spontaneous conversion to sinus rhythm approximately 10 minutes after initiation.

**Figure 3:**
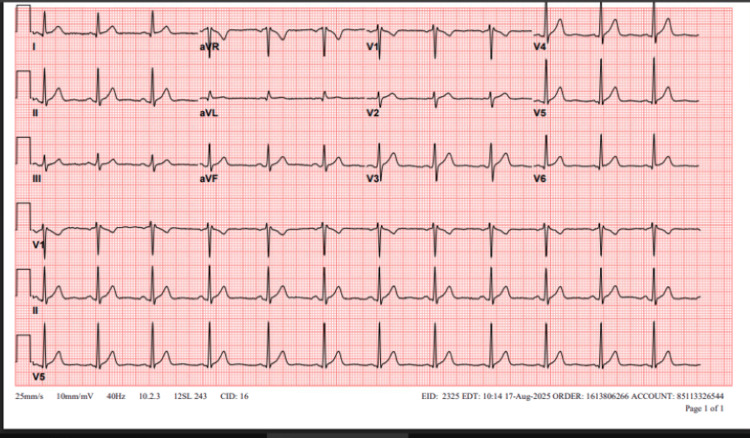
Repeat electrocardiogram (ECG) after conversion to sinus rhythm

**Figure 4:**
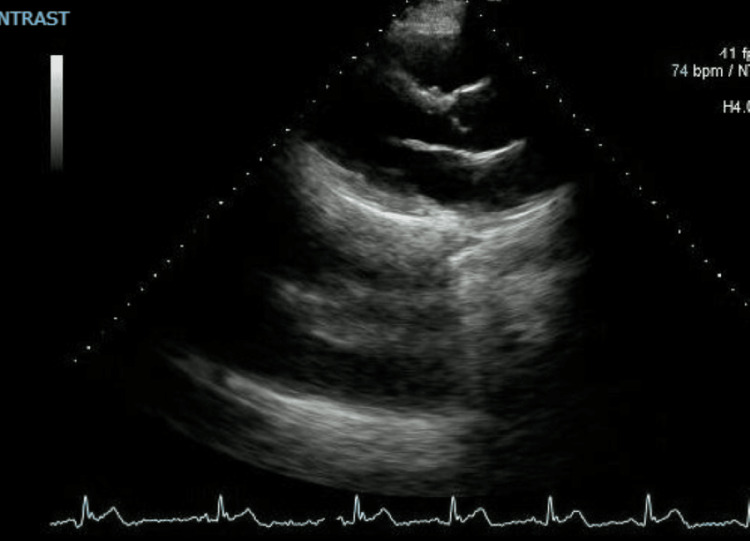
Transthoracic echocardiogram of the patient

The patient denied any alcohol or recreational drug intake, further evaluation showed normal thyroid function tests, and a repeat ECG (Figure 3) showed normal sinus rhythm. Blood alcohol concentration and urine drug screen were, however, not done. Transthoracic echocardiogram revealed only trace mitral and tricuspid regurgitation with no other structural abnormalities (Figure 4).

The patient remained in stable sinus rhythm during 24 hours of telemetry monitoring. Our Cardiology team deemed him fit and recommended discharge with outpatient follow-up. Given his CHA₂DS₂-VASc score of 0, he was discharged without anticoagulation or rate control medications, with plans for outpatient Holter monitoring and cardiology follow-up. The patient was, however, lost to follow-up despite multiple attempts to get him to return for further assessment.

## Discussion

This case presents a rare but clinically significant scenario: new-onset AF in a healthy young adult without identifiable risk factors, occurring after a bee sting and therapeutic administration of epinephrine.

AF is uncommon in individuals under the age of 40 in the absence of structural heart disease. When it does occur, reversible secondary causes should be carefully explored. These include thyroid dysfunction, electrolyte imbalances, infections, stimulant or substance use (such as alcohol or recreational drugs), and acute stress. In addition, sympathomimetic medications, including epinephrine and inhaled beta-agonists, are known to provoke arrhythmias in susceptible individuals [4].

Epinephrine is a potent alpha and beta-adrenergic agonist. Its beta-1 effects increase heart rate and atrioventricular node conduction, which may trigger supraventricular arrhythmias. While epinephrine remains life-saving in the context of anaphylaxis, transient AF has been documented in several case reports following therapeutic doses [5]. In one such report by Park and Khan, a young adult developed transient AF shortly after receiving both intramuscular and intravenous epinephrine for anaphylaxis, with spontaneous resolution after monitoring [6]. Another case by Jung et al. described inappropriate IV epinephrine administration in the prehospital setting, leading to hemodynamically unstable AF, highlighting the risks associated with dosage and route of administration [7].

Bee venom itself may also play a role in cardiovascular complications. It contains biologically active enzymes and peptides such as melittin, apamin, and phospholipases, which have vasoactive and neurotoxic properties. Some components can act directly on cardiac ion channels or provoke a systemic allergic response, leading to autonomic dysregulation. While cases of venom-induced arrhythmias have been documented, they are typically associated with more severe systemic reactions, such as Kounis syndrome, an allergic coronary vasospasm, or infarction [8].

In this patient, there was no clinical or echocardiographic evidence of myocardial ischemia or allergic myocarditis, and his symptoms were not consistent with coronary involvement. The timing of symptom onset in close proximity to epinephrine administration, combined with spontaneous resolution after rate control and the absence of structural or metabolic abnormalities, strongly supports epinephrine as the likely trigger.

However, the role of the body’s endogenous stress response to an allergic insult should not be overlooked. The physiologic release of catecholamines during an allergic reaction may have a synergistic effect when exogenous epinephrine is administered, particularly in young individuals with lower sympathetic tone thresholds. This may help explain why arrhythmias occur even in patients with no underlying cardiac history or risk factors. Ethanol level and urine toxicology were not obtained during the patient’s admission, and while these investigations could have helped to rule out alternative contributors to AF with rapid ventricular response, the clinical presentation and temporal relationship between the anaphylactic episode, epinephrine administration, and onset of arrhythmia support our conclusion. Nevertheless, the absence of these laboratory studies represents a limitation of this report.

While the arrhythmia in this case was transient and resolved with standard rate control therapy, it underscores the importance of close monitoring after epinephrine administration, especially in patients with persistent symptoms, palpitations, or signs of hemodynamic instability.

## Conclusions

This case highlights the importance of recognizing AF as a potential transient complication in young, otherwise healthy individuals following bee stings and epinephrine administration. Although epinephrine remains the gold standard for anaphylaxis treatment and should not be withheld due to arrhythmic risk, clinicians should remain vigilant for possible cardiac complications. Routine ECG monitoring, especially when patients report palpitations or tachycardia after treatment, can aid early recognition and management. Further studies are warranted to better understand the interaction between allergic reactions, autonomic activation, and epinephrine-induced arrhythmogenesis.
